# Diversity of Child and Family Characteristics of Children with Hearing Loss in Family-Centered Early Intervention in The Netherlands

**DOI:** 10.3390/jcm11082074

**Published:** 2022-04-07

**Authors:** Rosanne B. van der Zee, Evelien Dirks

**Affiliations:** 1 The Dutch Foundation of the Deaf and Hard of Hearing Child (NSDSK), 1073 GX Amsterdam, The Netherlands; edirks@nsdsk.nl; 2 Department of Psychology, Utrecht University, 3584 CH Utrecht, The Netherlands

**Keywords:** early intervention, hearing loss, language, characteristics

## Abstract

Background: Family-centered early intervention (FCEI) for children with hearing loss (HL) supports caregivers to promote their children’s language development. To provide FCEI services that are relevant and accessible to meet diverse needs, insight into the characteristics of children with HL is important. In the current study, various characteristics of children with HL and intervention-related factors are examined in relation to spoken language outcomes. Methods: Child and family characteristics, language outcomes and data on intervention were extracted from FCEI records for 83 children. Family involvement ratings were obtained from EI providers. Relations between characteristics, intervention, family involvement and language outcomes were analyzed and predictors for children’s language outcomes were investigated. Results: The characteristics of children with HL in FCEI are very diverse. Family involvement and the occurrence of additional disabilities were predictive for children’s receptive and expressive language abilities; the start of FCEI was not. Maternal education was predictive for expressive language outcomes only. Conclusions: The current study showed the diversity in characteristics of children with HL and their families in the degree of HL, etiology, cultural background, home language, family involvement and additional disabilities. We conclude that ’one size does not fit all’, and FCEI programs should acknowledge the unique strengths and challenges of every individual family.

## 1. Introduction

Family-centered early intervention (FCEI) for children with hearing loss (HL) aims at supporting caregivers to promote their children’s language development. FCEI is a family–professional partnership which builds on family strengths while taking different lifestyles, cultures and environments into account [1,2]. FCEI for children with hearing loss acknowledges that caregivers have a vital role in promoting their child’s development and well-being. The provision of timely FCEI for children with HL is associated with better language outcomes [3,4,5,6]. For children with hearing loss, the provision of FCEI has been recommended as the best practice by the Joint Committee in Infant Hearing [7] and is reinforced by the international consensus statement [2].

Family involvement is a foundational principle in FCEI [2,8]. Families, in general, are experts on their child and their involvement in FCEI has been linked to both child and caregiver-related outcomes. The positive effect of family involvement in FCEI on children’s language outcomes has been reported by several studies [9,10,11,12]. If caregivers participate regularly and actively in intervention sessions, make good adjustments to their child’s HL, advocate for their child and are effective conversational partners, language abilities increase. In addition, the increased involvement of fathers of children with HL in FCEI has been linked to higher levels of paternal self-efficacy [13,14].

In order to provide families with the opportunity to be involved in FCEI in a manner that suits their beliefs and needs, insight into the characteristics of children with HL and their families is of great importance. 

### 1.1. Diversity of Children and Families in FCEI

Children and their families who are enrolled in FCEI form an extremely heterogeneous group, varying greatly in terms of hearing loss-related factors [15], cultural and linguistic background [16] and the occurrence of additional disabilities [17,18]. All these factors may impact the family’s needs and the children’s developmental progress. For example, in FCEI programs, children with various degrees of HL are enrolled, which impacts the type of hearing amplification they use. Children with moderate HL often use hearing aids, while children with profound HL often wear a cochlear implant. The degree of HL negatively affects a child’s language outcomes. Studies comparing children with milder HL to those with more significant HL reported lower language skills in the latter group [19,20,21]. Therefore, early intervention (EI) providers should be aware of the potential effect of the severity of HL on a child’s developmental outcomes. Another important characteristic, which may vary between DHH children, is the age of enrollment in FCEI. Timely intervention (before 6 months of age) results in better language outcomes [4,5,6,8,20,21,22]. In addition, the dosage of intervention varies between families [22,23] and is positively related to language outcomes as well [23].

Childhood hearing loss is an etiologically heterogeneous condition, caused by genetic and/or environmental factors. Reports on the etiology of HL indicate that a genetic factor is responsible for the hearing loss in approximately 50% of the children with HL [24,25,26], of which 54% are estimated to be non-syndromic and 46% syndromic [26,27]. An acquired factor is reported for approximately 25% of the children with HL, including meningitis, cytomegalovirus (CMV) and risk factors such as hypoxia during birth and prematurity. For the remaining 25–30% of the children, the etiology is reported to be unknown. Knowledge of the etiology of HL enables the EI provider to anticipate and monitor possible risk factors associated with the child’s HL [26].

Besides the hearing loss, a substantial number of children have one or more additional disabilities. Around 20 to 40% of the children with HL cope with additional disabilities such as autism, blindness and developmental delays [18,28]. These additional disabilities could impact the development of children. Studies showed that having an additional disability negatively affected language outcomes in children with HL [21,29], and that the type of disability mattered [30,31]. However, early identification of additional disabilities in a child with HL could contribute to appropriate interventions for the child’s needs and could potentially improve developmental outcomes [32].

EI providers need to collaborate with families of different socioeconomic backgrounds. The educational level of caregivers might impact their ability to access information that is relevant for raising a child with HL. Studies reporting on the outcomes of children with HL, including children with cochlear implants and/or children with mild to moderate-severe HL, reported lower language abilities in children from families with a low educational level [19,21,33]. However, a recent meta-analysis about the impact of the family environment on the language development of deaf children with cochlear implants found no evidence of the relationship between parental education and language development of the child [34]. 

The increasing cultural and linguistic diversity and multilingualism of children with HL and their families is an important consideration in FCEI programs [2,16,35,36]. A family’s values and beliefs might influence their perspectives on parenthood, raising a child with HL, relationships with EI providers and involvement in the FCEI program. It is thus important to understand and be responsive to cultural diversity [2,8,36] and to be aware of the cultural diversity of families within FCEI programs. In addition, the hearing status of caregivers is of importance. Around 4.4% of children with HL have one or more caregivers with HL (1.1% one parent with HL and 3.3% both parents with HL [15]). The needs of these families, who have experience with HL, might differ from those without prior experience with HL. Children born into families with one or both parents having HL had better vocabulary outcomes [21]. This might be due to their access to a rich sign language environment and/or exposure to effective communication strategies [37]. Children with HL learn to communicate in spoken language, sign language or a combination of both. This may vary according to their home environment, communication partners and age [35]. In some families, multiple spoken languages are used, while in others there is only one spoken language which may or may not be combined with the use of signs.

There is a lot of diversity in children with HL and their families. FCEI programs should address this diversity to ensure that all children benefit from intervention. While many studies have reported on specific aspects of diversity or studied developmental outcomes in subgroups of children, it is of relevance to gain insight into the characteristics of an entire population of children who are enrolled in an FCEI program. These insights might contribute to the adjustment of FCEI programs to the various needs of children with HL and their families.

### 1.2. Current Study

The current study will report on the characteristics of an entire population of children with HL in a Dutch FCEI setting. In the Netherlands, the trajectory from hearing screening to FCEI is consolidated over the years. Participation rates for screening, audiological assessments and enrollment in FCEI are high [38]. Of all newborns, 99.7% participate in the Dutch newborn hearing screening program and 95% of the children with a referral for diagnostic testing follow-up on this [38]. The hearing screening program in the Netherlands has high follow-up rates in comparison to other countries [39,40,41,42]. One study (with a small sample size) reported that 87% of all children with a diagnosis of HL enroll in FCEI [43]. Based on these numbers, it is assumed that the population of children with HL in the current study is representative of the Dutch population of children with HL. Studies on the characteristics of children with HL in FCEI in the Netherlands are lacking. 

The main aim of the current study is to gain insight into the characteristics of Dutch children with HL and their families who are enrolled in FCEI. To provide FCEI services that are relevant and accessible to meet diverse needs, a first step is to achieve insight into the characteristics of the target group. In addition, we will examine which characteristics are predictive of spoken language outcomes, focusing on family involvement, degree of HL, additional disabilities, maternal educational level and intervention-related factors. Based on the previous findings reported above, we expect that family involvement, the absence of additional disabilities, higher maternal educational level and less severe HL will positively predict language outcomes. 

## 2. Materials and Methods

### 2.1. Participants

Participants in this study were children with bilateral hearing loss who are enrolled in the FCEI program of the Dutch Foundation of the Deaf and Hard of Hearing Child (NSDSK). The NSDSK is the primary provider of FCEI for children with HL and their families in the north-west region of the Netherlands. In this region, the total number of births is around 30,000 a year [44]. All participants were born in a birth cohort of three years (2014–2016). In this period, 101 DHH children were registered for FCEI at the NSDSK. Based on the prevalence of HL in newborns in the Netherlands [38] and the birth rate in the region, it is assumed that approximately 110 children with HL were born in the working region of the NSDSK in these years. This means that roughly 92% of all children with HL in this region were referred to the NSDSK FCEI program. 

**Figure 1:**
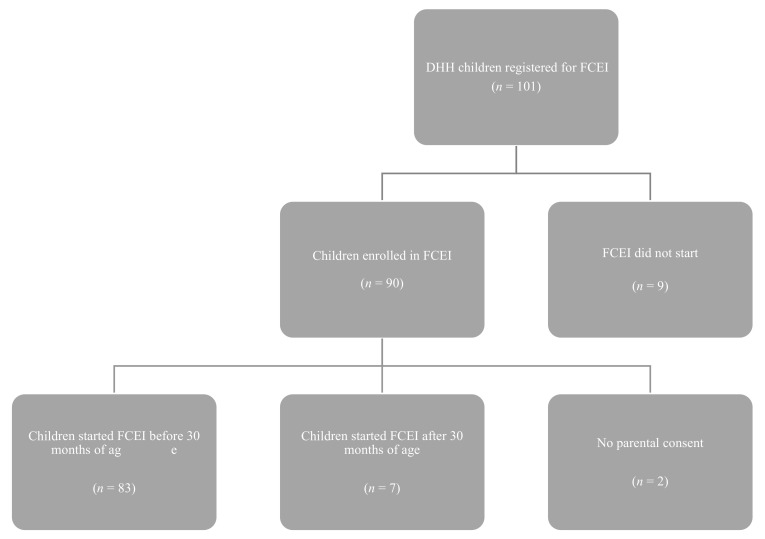
An overview of the inclusion of the participants in this study.

For 9% of the 101 children FCEI did not start, because parents declined the intervention, children were enrolled at another FCEI center (in another region) or for unknown reasons. Eventually, 92 children were enrolled in FCEI. Children were excluded from the current study if there was no parental consent (*n* = 2), or if FCEI had been started after the child was 30 months of age (*n* = 7) because language outcomes at the age of 30 months were used in this study. For 5 children who started after the age of 30 months, this was due to an unclear diagnosis of permanent hearing loss for a long time (e.g., hearing loss was progressive, hearing loss was partly conductive or hearing loss was around 40 dB). This led to a total number of 83 participants. An overview of the inclusion of the participants is presented in Figure 1. 

#### Family-Centered Early Intervention 

In the Netherlands, there are seven organizations that provide FCEI for children with HL and their families. Each organization provides FCEI in a different region of the country. Children with permanent bilateral hearing loss ≥40 dB are eligible for FCEI in the Netherlands. The aim is to start FCEI before the child is 6 months of age, in line with the 1-3-6-guidelines [7]. Children are usually enrolled in FCEI until the age of four, i.e., the age at which most children in the Netherlands start formal school. An EI provider is assigned to each family during this trajectory in order to support and guide parents in promoting their child’s language and social-emotional development. Each family has one dedicated EI provider who serves as a point of contact around the child with HL. This EI provider provides caregivers with information about hearing loss, hearing technology, communication, language development, social-emotional development and coordinates the FCEI around the child and family. In addition, the EI provider provides social and emotional support to caregivers to promote their well-being.

Early intervention sessions from the NSDSK are usually at home, at least once per month and take approximately 90 min. In addition to the home visits by the early interventionist, other EI providers will support the families at home or at the FCEI center, for example, sign teachers, speech and language therapists and social workers. From the age of 18 months, intervention takes place at the center as well. For example, children with HL can join an intervention group. This more child-directed intervention is in addition to the intervention at home. The FCEI center also provides courses to the caregivers, such as sign lessons, courses to promote children’s language development and courses on storybook reading [45]. 

### 2.2. Procedure

In order to gain insight into the diverse group of children with HL and their families, characteristics of the children were extracted from their FCEI records. Information on a child’s hearing loss (degree, etiology, amplification), occurrence of additional disabilities, languages used at home and family characteristics (siblings, type of family, hearing loss within the family, cultural background, maternal education) were used for the current study. Data were extracted from all children whose parents gave informed consent to use these data for research purposes. Language use in the family was registered at the start of the FCEI. EI providers were asked retrospectively to classify the communication mode of the families and family involvement. Standardized language tests were assessed regularly to monitor the development of children with HL and were extracted from the FCEI records. 

Data on the dosage of intervention that children received were obtained from the FCEI records as well. For this study, the total number of FCEI sessions per child per month was calculated up to the age of 18 months. The total number of FCEI sessions per child was then divided by the length of time children were enrolled in FCEI. For 14 children these data were missing, because they were enrolled in FCEI after the age of 18 months. We only calculated the FCEI sessions before the age of 18 months, because after this age children could attend the intervention groups at the EI center in addition to the house visits. When children attend the intervention groups (two partial days a week), the frequency of house visits decreases. In the current study, 73% of the children visited these intervention groups.

### 2.3. Measures

#### 2.3.1. Family Involvement

The involvement of the families was assessed by using a Dutch translation of the Family Participation Rating Scale developed by Moeller [9]. The family’s dedicated EI provider was asked to rate family involvement in FCEI retrospectively on a 5-point scale (1 = limited participation, 2 = below average participation, 3 = average participation, 4 = good participation, 5 = ideal participation). EI providers were asked to base their score on family adjustment to the HL, participation in FCEI sessions, effectiveness of communication with the child and advocacy for the child. Extensive descriptions for each category were available to EI providers when ratings were given [9]. Ratings were assigned by EI providers who had very extensive contact with the families during the whole FCEI trajectory. 

#### 2.3.2. Language 

The receptive language skills of all children were assessed with the Dutch Schlichting Receptive Language Test [46]. In this test, children are asked to perform simple tasks with toy animals, like ‘put the monkey on the house’. Expressive language skills were assessed with the Dutch Schlichting Expressive Language Test [47]. In this test, syntactic structures are elicited in a playful manner. These norm-referenced language tests are commonly used in the Netherlands and have standardized Q-scores with a mean of 100 and an SD of 15. For 11 of the DHH children in our sample, Dutch was not one of the spoken languages used at home. These children were excluded from analyses where language performance was taken into account. Since the language abilities of the children were only assessed in Dutch, the scores of these children were not reliable. Language was assessed, by speech and language therapists or a clinical linguist, at the age of 30 months, with a range from 24 to 36 months. 

### 2.4. Data Analysis

To analyze the relationship between child and family characteristics, intervention-related factors and language outcomes, a one-tailed Spearman’s correlation analysis was performed. Multiple regression models were used to explore the predictors for expressive and receptive language ability at the age of 30 months. The variable ‘maternal education’ was transformed into a dichotomous variable for these analyses. Table 1 describes the coding of the independent variables included in the models.

## 3. Results

### 3.1. Characteristics of Children with HL in FCEI

The demographic characteristics of the children with bilateral HL and their families are presented in Table 2. Most children had moderate hearing loss and consequently the use of hearing aids was most frequent. At the age of two, additional disabilities were reported in 20% of the children. At the start of FCEI, 39% of the families used another language in addition or instead of Dutch. Approximately 20 different languages were being used in the various families. Polish, Arabic, Spanish, Turkish and English were the most common. Two of the families used Dutch Sign Language at home. They were already familiar with sign language because parents or siblings of the child had hearing loss as well. In total, 19% of the children were raised in a family where a parent(s) or sibling(s) had hearing loss. In 81% of the families, hearing loss did not occur within the family. In addition to language use, the communication mode within the families during FCEI was inquired about. Most families used a combination of spoken language and signs (64%).

**Figure 2:**
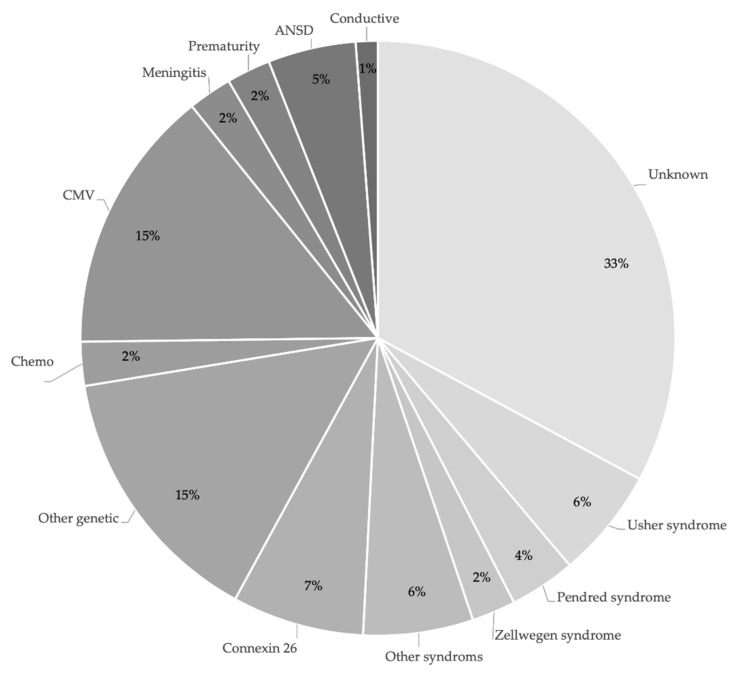
Etiology of hearing loss. (ANSD = Auditory Neuropathy Spectrum Disorder/CMV = Cytomegalovirus).

In order to gain more insight into the diverse characteristics of the DHH children, we looked into the etiology of the hearing loss in more detail. If parents provided the EI provider with information on the etiology of a child’s hearing loss, this was registered in the FCEI record. Results on etiology are presented in Figure 2. The cause of hearing loss was, for most children, genetic (40%). Many of these children had a syndrome associated with hearing loss. Hearing loss was acquired in 26% of the children, due to a CMV infection, meningitis, chemotherapy, prematurity, ANSD or a conductive hearing loss. Information on the etiology of hearing loss was unknown for 33% of the children. 

### 3.2. Intervention-Related Factors

In Table 3, details on intervention-related factors are presented. From the total population, 61% of the children were enrolled in FCEI before the age of 6 months. At this age, 59% of the children had started wearing hearing aids. Within the group of children that were diagnosed with hearing loss before the age of 3 months, 85% were enrolled in FCEI at the age of 6 months. Reasons for late enrollment in FCEI were: the diagnosis of permanent hearing loss was unclear for a long time (e.g., hearing loss was progressive, hearing loss was partly conductive or hearing loss was around 40 dB) (39%), severe medical problems (14%), born in another country (11%), parents declined FCEI at first (7%), non-congenital HL (14%), referral from another FCEI organization (7%) and unknown reasons (8%). Table 2 shows that the number of FCEI sessions before the age of 18 months varied greatly between the families. Some of the families received FCEI once a month and others weekly. Most families (54%) received between one and two sessions per month, 25% more than twice a month and 22% received FCEI less than once a month. Mean ratings on family involvement and language scores of the children are presented in Table 2. Most families received an involvement rating from average to ideal participation (80%), which resulted in a mean score between average and good participation. Involvement ratings were missing for six children, because FCEI providers indicated that they could not provide a reliable rating. 

The mean of the expressive language scores was within the normal range; the mean of the receptive language abilities of the children was 1 SD below the mean. Both language outcomes had a very wide range. Receptive language scores were missing for 26 children, and expressive language scores for 32 children. The main reasons for the missing language outcomes were short enrollment in FCEI or the low language abilities of the children, often related to additional disabilities. 

### 3.3. Correlations between Language Outcomes and Characteristics

To explore the relationships between language outcomes, family involvement, intervention and various demographic variables, Spearman correlations were calculated (Table 4). Language outcomes were related to family involvement and disability status. Expressive language outcomes were also related to the start of FCEI. Children that were enrolled before the age of 6 months had higher expressive language scores. Family involvement had a significant correlation with language outcomes, level of maternal education and number of FCEI sessions per month. In addition, there was a significant relationship between additional disabilities and language outcomes. Furthermore, the start of FCEI and the dosage of FCEI correlated with the degree of hearing loss. Children with more severe HL were enrolled earlier and received more FCEI sessions per month than children with less severe HL.

### 3.4. Predictors of Language Outcomes

To identify predictors of variance in language outcomes of the children at the age of 30 months, a multiple linear regression analysis was used. The degree of HL, start of FCEI and dosage of FCEI were initially included in the model to confirm that they were not significantly related to language outcomes. These variables remained non-significant and were removed from the final model. Four independent variables (family involvement, disability status, start of FCEI, maternal education) were entered into the final regression model. 

The overall model for receptive language was significant (F_−4,46_ = 4.05, *p* = 0.007) and explained 26.0% of the variance in receptive language skills of the children. Table 5 shows that family involvement and the occurrence of an additional disability in the child made a significant contribution to the model. The start of FCEI and maternal education were not significant. The model for expressive language was significant as well (F_−4,42_ = 5.58, *p* = 0.001), and explained 34.7% of the variance in expressive language skills of the children. Family involvement, the occurrence of an additional disability in the child and the level of maternal education made a significant contribution to the model (Table 6). The start of FCEI was not a significant predictor. 

Both receptive and expressive language outcomes were shown to increase when family involvement was higher (Table 5 and Table 6). The occurrence of additional disabilities decreased receptive and expressive language outcomes. A higher level of maternal education was found to contribute to the expressive language abilities of the child. The start of FCEI did not seem to be related to language outcomes in these models. 

## 4. Discussion

FCEI for children with HL aims to support caregivers in promoting their child’s development by addressing the beliefs and needs of the whole family. By supporting and guiding families, it is important to consider their strengths and characteristics. Previous studies indicated that children with HL vary in child and family characteristics and intervention-related factors [19,21,22,23,24,30,48]. 

The current study aimed to provide insights into the diverse population of Dutch children with HL and their families in FCEI. In addition, predictors of children’s language outcomes were examined. The findings demonstrated that children in FCEI form a heterogeneous group, not only in hearing-related characteristics but in family characteristics and intervention-related factors as well. Furthermore, family involvement and the occurrence of additional disabilities were predictive for children’s receptive and expressive language abilities. 

### 4.1. Diversity of Children and Families in FCEI

The current study confirms that the characteristics of children with HL and their families are very diverse. When looking into the etiology of the children’s HL, a genetic cause was reported in 40% of the children, with connexin26 being the most prevalent. An acquired factor was found in 26% of the children, with CMV being the most prevalent. This is in line with larger studies reporting on the etiology of HL in children [24,25,26]. The etiology of HL may impact the developmental progress of children and is of relevance for supporting caregivers in raising their child. Additional disabilities were reported in 20% of the children, in line with findings from the study of Ching et al. [20], but in contrast to other studies reporting a higher number [18,28]. Since the children in the current study were only 30 months of age, presumably certain additional disabilities may not have appeared yet. 

In the current study, 11% of the children had one or more parents with HL and 12% of the children had one or more siblings with HL. Children who grow up with other family members with HL can benefit from their experience and knowledge regarding HL. Caregivers who have more children with HL may have different needs than parents who have no prior experiences in raising a child with HL. 

Families with various levels of education were enrolled in the FCEI program. The maternal educational level was positively related to family involvement and the predicted expressive language abilities of the children at the age of 30 months. It would be relevant to explore which strategies might improve the involvement of families with a low educational level. The current study showed that the population of children with HL is diverse in language use and cultural background as well. More than 25% of the children grew up in a multilingual family with 20 different languages in total. In 13% of the families, Dutch was not spoken at all, and interpreters were sometimes needed. It demands a lot from EI providers to adapt the intervention to families from all these different backgrounds.

### 4.2. Language Outcomes

Language outcomes for this diverse population of 30-month-old children with HL were a standard deviation below the mean for receptive language and were in the normal range (low average) for expressive language. These findings are in line with other studies regarding language outcomes of children with HL at the same age [19,21,33]. Contrary to expectations and findings reported by others [6,23,49], it turned out that the start of FCEI, before or after 6 months of age, was not predictable for language outcomes in our study. The mean age of enrollment was 7 months, and 61% of the children were enrolled before they were 6 months old. Furthermore, seven children that were enrolled in FCEI after the age of 30 months were excluded from the current study. These are possible explanations for these findings. The dosage of FCEI sessions was also not related to language outcomes. This is in contrast to recent findings from Wiggin et al. [23] who reported a relation between the frequency of FCEI sessions and children’s vocabulary scores. In the Wiggin study, a parental inventory was used to assess language abilities, while in the current study, both receptive and expressive language abilities were assessed by a speech and language therapist. In addition, the time between the FCEI sessions and language outcomes was not comparable between studies. This might impact the differences in findings. Furthermore, the number of FCEI sessions in the current study was extracted from registrations in the FCEI records, instead of reported on by parents. 

In line with other studies [21,28,30] and our expectations, the occurrence of additional disabilities in children with HL negatively affected their language outcomes. The sample size of the current study did not allow for detailed analyses on the type of additional disability in relation to language abilities.

Family involvement is essential in FCEI and is promoted by EI providers. In accordance with earlier findings [9,11,49], family involvement was shown to be predictive of language outcomes in children with HL. Although 80% of the families in the current study were found to be average to highly involved, it is recommendable to be alert on those families who are less involved. 

### 4.3. Limitations and Directions for Future Research

There are limitations of the current study that should be considered. First, language outcomes were not available for all children within the FCEI program. For some of the children, especially children with an additional disability, the language level of the used tests was too high. If possible, other language tests were used to assess the language ability of these children, but these were not included in this study. Almost 40% of the children in this study used one or more languages other than Dutch at home. For children from multilingual families, reliable testing is very complicated and language assessments in other languages are not available. Language ability is often only assessed for spoken Dutch, not for other spoken languages or Dutch Sign Language. In order to monitor the development of the total population of children with HL, reliable testing for multilingual children is essential. 

Secondly, family involvement was only reported by the primary EI provider of the family. These EI providers were dedicated to the families for several years and knew the families and children quite well. The EI providers were thus not blinded for the children’s language outcomes which might have impacted their judgement. In addition, when EI providers were not able to establish a strong relationship with a family, this could also result in a judgement of poor family involvement. It would be interesting for future research to ask the families themselves to report on their involvement in FCEI. In a recent study among Dutch fathers and mothers with a child with HL, parents reported on their own involvement in FCEI [14]. Family involvement showed to be related to parental self-efficacy in fathers but not in mothers. 

### 4.4. Implications for FCEI 

With this study, we expand the knowledge on characteristics and outcomes of diverse populations of children with HL. One of the strengths of the current study is the representativeness of the sample of children with HL in FCEI. The Dutch hearing screening program shows high participation and follow-up rates, and the number of children enrolled in the NSDSK FCEI program is likely to include most children with HL in the region. While in many studies the group of mothers with low educational levels is underrepresented, the educational levels of mothers in the current study are comparable to the general educational level of Dutch mothers, with even a slightly higher number of mothers with a low educational level. 

The current study showed the diversity in characteristics of children with HL and their families in terms of severity of HL, etiology, cultural background, language use, communication mode, family involvement and additional disabilities. We therefore conclude that ‘one size does not fit all’, and FCEI programs should acknowledge the unique strengths and challenges of every individual family. Expanding the knowledge of EI providers on the diverse population of children with HL and the characteristics that may impact their development will benefit children’s outcomes.

## Figures and Tables

**Table 1:** Description of the coding of the independent variables in the multiple regression models.

Independent Variable	Coding Variable
Family involvement	Continuous variable
Disability status	0 = no additional disability 1 = occurrence of additional disability
Maternal education	0 = low/medium level of education 1 = high level of education
Degree of hearing loss	Continuous variable
Start of FCEI	0 = FCEI started after 6 months of age 1 = FCEI started before 6 months of age
Dosage of FCEI	Continuous variable: number per month

**Table 2:** Characteristics of the participants and their families.

	*n*	%
Gender		
Male	44	53.0
Female	39	47.0
Hearing loss ^1^		
Moderate (40–60 dB)	47	56.6
Severe (61–80 dB)	10	12.0
Profound (>80 dB)	26	31.3
Type of amplification		
Hearing aid	49	59.0
Cochlear implant (bilateral)	24	28.9
Bone conduction hearing aid	4	4.8
Hearing aid and cochlear implant	6	7.2
Additional disabilities		
None	66	79.5
Developmental delay	5	6.0
Vision impairment	3	3.6
Vision impairment and developmental delay	2	2.4
Autism spectrum disorder	2	2.4
Other	5	6.0
Family		
Single-parent family	6	7.2
Two-parent family	77	92.8
Hearing loss in the family		
One parent with hearing loss	7	8.4
Two parents with hearing loss	2	2.4
Sibling(s) with hearing loss	10	12.0
Maternal education ^2^		
Low	12	14.5
Medium	20	24.1
High	44	53.0
Unknown	7	8.4
Language use in the family at the start of FCEI		
Dutch only	51	61.4
Dutch in combination with other spoken language(s)	19	22.9
Dutch in combination with sign language	2	2.4
Other spoken language(s)	11	13.3
Communication mode with the child during FCEI		
Sign language only	1	1.2
Spoken language(s) with signs/sign language	53	63.9
Spoken language(s) only	22	26.5
Unknown	7	8.4
Cultural background ^3^		
Dutch	66	79.5
Western	8	9.6
Non-Western	8	9.6
Unknown	1	1.2

^1^ Degree of hearing loss was determined at the start of FCEI from results of Auditory Brainstem Response (ABR). ^2^ Low = primary education, intermediate preparatory vocational education or level 1 of secondary vocational education; medium = secondary vocational education, senior general secondary education or pre-university education; high = higher vocational education or academic education. Maternal education in our sample is quite comparable to the educational level of mothers in the Netherlands. The number of mothers with a high and low educational level is slightly higher in our sample than in the general Dutch population of mothers with a child between 0 and 2 years of age. (Source: CBS statline, [44]). ^3^ Western = originating from a country in Europe (excluding Turkey), North America and Oceania, or from Indonesia or Japan/non-Western = originating from a country in Africa, South America or Asia (excluding Indonesia and Japan) or from Turkey.

**Table 3:** Details on intervention-related factors (mean, SD, range and number of participants).

	Mean	Range	*n*
Age at start of amplification (in months)			
Hearing aid users	8.98 (7.61)	2–28	49
Cochlear implant users	14.21 (4.26)	11–28	24
Bone conduction hearing aid users	4.75 (4.35)	1–11	4
Hearing aid and cochlear implant users	8.17 (4.71)	2–13	6
Age at enrollment in FCEI (in months)	7.19 (7.49)	1–28	83
FCEI sessions per month ^1^	1.64 (0.74)	0.54–4.86	69
Family involvement	3.68 (1.19)	1–5	77
Receptive language score	84.12 (17.94)	55–120	57
Expressive language score	91.22 (11.95)	71–123	52

^1^ 14 children were not enrolled in FCEI before the age of 18 months.

**Table 4:** Correlations between language outcomes and characteristics of the children with HL.

	1	2	3	4	5	6	7	8
1. Receptive language	-	-	-	-	-	-	-	-
2. Expressive language	0.72 **	-	-	-	-	-	-	-
3. Family involvement	0.26 *	0.26 *	-	-	-	-	-	-
4. Disability status	−0.48 **	−0.39 **	0.07	-	-	-	-	-
5. Maternal education	0.05	−0.19	0.38 **	−0.02	-	-	-	-
6. Degree of hearing loss	−0.02	−0.15	0.0	0.03	−0.19	-	-	-
7. Start of FCEI	0.15	0.24 *	−0.06	−0.15	−0.00	0.22 *	-	-
8. Dosage of FCEI	0.08	0.18	0.36 **	−0.01	0.17	0.45 **	−0.14	-

* *p* < 0.05, 1-tailed. ** *p* < 0.01, 1-tailed.

**Table 5:** Multiple regression predicting children’s receptive language outcomes at the age of 30 months.

Child and Family Characteristics	Standardized Coefficient	Unstandardized Coefficient	*t* Test Value	*p*
Family involvement	0.29	4.37	2.22	0.032
Disability status	−0.41	−19.30	−3.19	0.003
Start of FCEI	0.13	4.65	0.99	0.329
Maternal education	−0.08	−3.11	−0.64	0.528

**Table 6:** Multiple regression predicting children’s expressive language outcomes at the age of 30 months.

Child and Family Characteristics	Standardized Coefficient	Unstandardized Coefficient	*t* Test Value	*p*
Family involvement	0.41	3.99	3.07	0.004
Disability status	−0.37	−11.45	−2.93	0.005
Start of FCEI	0.20	5.02	1.60	0.118
Maternal education	−0.35	−8.83	−2.61	0.012

## Data Availability

Data are not publicly available because we do not have consent in favor of public access of the parents of the subjects involved.
